# Constructing ZIF-8 derived C–ZnS/ZnMoO_4_@MoS_2_ and C–ZnS/MoS_2_ nanocomposites using a simple one-pot strategy to enhance photocatalytic degradation activity†

**DOI:** 10.1039/c9ra06591a

**Published:** 2019-10-31

**Authors:** Yi-Wei Cui, Hai-Huan Zhang, Shi-Yong Yu

**Affiliations:** Inner Mongolia Key Laboratory of Mongolian Medicine Chemistry, Inner Mongolia Key Laboratory of Coal Chemistry, School of Chemistry and Chemical Engineering, Inner Mongolia University Hohhot 010021 Inner Mongolia China syyunano@imu.edu.cn

## Abstract

Efficient C–ZnS/ZnMoO_4_@MoS_2_ and C–ZnS/MoS_2_ nanocomposite photocatalysts, using ZIF-8 derived C–ZnO as a precursor were successfully synthesized using a simple one-pot procedure. This is the first application that involves transforming ZIF-8 into C–ZnMoO_4_ for photocatalysis. The C–ZnS/ZnMoO_4_@MoS_2_ and C–ZnS/MoS_2_ heterostructures were characterized by X-ray diffraction, UV-vis, X-ray photoelectron spectroscopy, electrochemical impedance spectroscopy, photocurrent measurements, scanning electron microscopy and transmission electron microscopy. The ZM2 sample of C–ZnS/ZnMoO_4_@MoS_2_ exhibited enhanced photocatalytic activity of about 2.9 times as high as that of ZIF-8 derived C–ZnO in the reduction of tetracycline hydrochloride, and also showed obvious photocatalytic activity 1.81 and 3.33 times as high as that of a ZM3 sample of C–ZnS/MoS_2_ and ZIF-8 derived C–ZnO in the degradation of RhB, respectively. The improved photodegradation activity is a result of the heterogenous structure and the tighter contact between C–ZnS and C–ZnMoO_4_ compared with the physical contact of general heterogenous photocatalysts. The C–ZnS/ZnMoO_4_@MoS_2_ heterostructure photocatalyst is expected to be a new type of nanomaterial for the degradation of pollutants from wastewater.

## Abstract 2

C-doped ZnS/ZnMoO_4_@MoS_2_ and ZnS/MoS_2_ were prepared using ZIF-8 as a precursor; a simple one-pot strategy was adopted to produce the catalysts with tighter contact.

## Graphical abstract

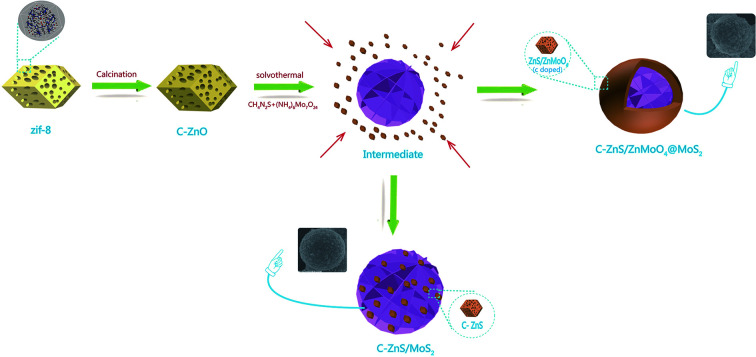

## 1. Introduction

Wastewater containing antibiotics has become a grave threat to human health and the environment.^1,2^ Tetracycline hydrochloride (TC-H) as the second most widely distributed antibiotic in the world has been extensively produced and used.^3,4^ However, due to the unique characteristic properties of TC-H, only 10–30% of TC-H can be metabolized by animals and human beings, and 70–90% is released into the water environment *via* discharge of wastewater effluent and animal manure after being heavily used in the pharmaceutical industry, animal husbandry and aquaculture, which causes serious water environmental problems.^5,6^

Among the treatments of tetracycline wastewater, photocatalysis is a simple,^7^ dependable and environmentally-friendly technology compared to traditional techniques, such as adsorption,^8^ microbial degradation^9^ or membrane separation.^10^ Compared with conventional photocatalysts, such as TiO_2_, C_3_N_4_ or ZnO,^11–13^ using MOFs as “precursors” or self-sacrificial frameworks to fabricate porous metal oxides as photocatalysts has been a research hotspot in recent years.^14,15^

Zeolite imidazolate frameworks (ZIFs) as a sub aggregation of MOFs are a kind of porous crystalline material in which imidazolate linkers are cross-linked to transition metal ions to form 3D tetrahedral frameworks.^16–19^ They have attracted extensive attention in various fields, such as heterogeneous catalysis,^20–23^ photocatalysis,^24^ sensing,^25^ gas adsorption^26–28^ and drug delivery due to their high surface area and high crystallinity as well as good chemical and thermal stability.^29^ However, as photocatalysts, most ZIFs cannot be excited by visible light because their band gaps are too wide to be excited: for example, ZIF-8 has a band gap of 5.1 eV.^30^ For the purposes of diminishing their band gaps and broadening their application in photocatalysis, MOFs can be used as precursors to directly prepare C-doped oxides and it has been proved that the prepared C-doped materials can expand the response to visible light and facilitate the absorption of visible light. Du *et al.* prepared porous C–ZnO by calcining ZIF-8 directly in air and they found that such ZnO showed improved photocatalytic activity compared to ZIF-8 or pure ZnO.^31^ Feng *et al.* prepared N–ZnO from the direct calcination of urea and a ZIF-8 mixture for RhB degradation, and found that such a material can enhance the visible-light harvesting ability and achieve a photocatalytic efficiency higher than that of ZIF-8 derived C–ZnO.^32^ However, reports about transforming ZIF-8 directly into heterojunctions are very scarce.

Molybdenum disulfide (MoS_2_) has been proven to be an excellent co-catalyst in photocatalytic activity by providing more active sites and inhibiting the recombination rate of electron–hole pairs. Thomas *et al.* reported active edge sites for electrochemical hydrogen production from MoS_2_.^33^ By contrast, ZnS and ZnMoO_4_ are both well-known photocatalysts, which have a wider band gap than MoS_2_. Such a combination can facilitate the separation of the electron–hole pairs by photoexcitation.^34,35^

Inspired by this, herein, we have adopted a one-pot route in which ZIF-8 derived C–ZnO was added to the raw materials for the synthesis of MoS_2_ and a three-phase heterojunction of C–ZnS/ZnMoO_4_@MoS_2_ and a two-phase heterojunction of ZnS/MoS_2_ could be finally obtained. It is worth mentioning that, because the C–ZnS and C–ZnMoO_4_ in the three-phase heterojunction were directly produced together from ZIF-8 derived C–ZnO in the same step, the contact between C–ZnS and C–ZnMoO_4_ will be tighter than the physical contact of general heterojunction photocatalysts, and this can facilitate the separation of the electron–hole pairs.

## 2. Experimental section

### 2.1 Materials

Zinc nitrate hexahydrate [Zn(NO_3_)_2_·6H_2_O ≥ 99.0%], absolute methanol (EtOH ≥ 99.7%) and ethanol (EtOH ≥ 99.7%) were purchased from Sinopharm Chemical Reagents Co., Ltd. 2-Methylimidazole [H-MeIM], thiourea (CH_4_N_2_S) and ammonium molybdate [(NH_4_)_6_Mo_7_O_24_·4H_2_O] were purchased from Aladdin (P. R. China). All chemicals were used without any further purification.

### 2.2 Preparation

#### 2.2.1 Preparation of zeolitic imidazolate framework-8 (ZIF-8)

ZIF-8 was synthesized according to previously reported studies.^36^ In a typical synthesis, 0.9852 g of 2-methylimidazole was dissolved in 15 ml of methyl alcohol and 0.8925 g of zinc nitrate hexahydrate [Zn(NO_3_)_2_·6H_2_O] was dissolved in 10 ml of methyl alcohol. After that 2-methylimidazole and zinc nitrate hexahydrate were mixed together and stirred at room temperature for 24 h. The sample was collected by centrifugation and washed several times with methyl alcohol and then dried at 50 °C overnight.

#### 2.2.2 Preparation of ZIF-8 derived C doped ZnO (C–ZnO)

ZIF-8 derived C–ZnO was obtained directly by calcining ZIF-8 powders at 380 °C for 2 h at a heating rate of 2 °C min^−1^.

#### 2.2.3 Preparation of ZIF-8 derived C-doped ZnS/ZnMoO_4_@MoS_2_ (C–ZnS/ZnMoO_4_@MoS_2_) named as ZM1,2,4, and C-doped ZnS/MoS_2_ (C–ZnS/MoS_2_) named as ZM3

In a typical preparation process of ZIF-8 derived C–ZnS/ZnMoO_4_@MoS_2_, 50 mg of ZIF-8 derived C–ZnO powder was added to 30 ml of deionized water and sonicated for 30 min to form a homogeneous dispersion. Then, 0.12 g of CH_4_N_2_S and 0.48 g of NH_4_MoO_4_ were added to the preceding solvent and stirred for 2 h. After that, the obtained solution was transferred to a 45 ml Teflon-lined autoclave and maintained at 200 °C for 24 h in an oven. The resulting samples were collected by centrifugation, and washed several times with deionized water and ethyl alcohol. In order to synthesize materials with different ratios of ZnS, ZnMoO_4_ and MoS_2_, a series of samples were prepared by adding different raw materials. In general, C-doped ZnS/ZnMoO_4_@MoS_2_ was produced from 50 mg of C–ZnO, 0.12 g of CH_4_N_2_S and 0.24 g of NH_4_MoO_4_ and named as ZM1; from 50 mg of C–ZnO, 0.12 g of CH_4_N_2_S and 0.48 g of NH_4_MoO_4_ named as ZM2; and from 25 mg of C-doped ZnO, 0.12 g of CH_4_N_2_S and 0.24 g of NH_4_MoO_4_ named as ZM4. C–ZnS/MoS_2_ was prepared by adding 50 mg of C–ZnO, 0.24 g of CH_4_N_2_S and 0.48 g of NH_4_MoO_4_ and named as ZM3.

### 2.3 Characterization of the photocatalysts

The crystallographic phase of the as-synthesized samples was determined with a Panalytical X-Pert X-ray diffractometer (XRD) with a scanning rate of 2.5° per min. The morphology and elemental compositions of the prepared photocatalysts were observed by a scanning electron microscope (SEM) equipped with an energy dispersive X-ray (EDX) spectrometer and transmission electron microscopy (TEM, JEM-2010). X-ray photoelectron spectroscopy (XPS) was performed on an ESCALAB250 instrument (Thermo VG Corp.) The UV-vis spectra were carried out on a spectrometer (Shimadzu UV-3600). The photoluminescence (PL) spectra of the samples were recorded on an Edinburgh Instruments FLS 920 spectrometer at an excitation wavelength of 350 nm.

### 2.4 Photocatalytic measurements

The photocatalytic activities of C–ZnS/ZnMoO_4_@MoS_2_ and C–ZnS/MoS_2_ were measured using the photodegradation of tetracycline hydrochloride and RhB in an aqueous solution under full spectrum light irradiation (320 nm < *λ* < 780 nm) using a 300 W xenon lamp. Typically, in the degradation process of tetracycline hydrochloride: 15 mg of catalyst was dispersed in 30 ml of tetracycline hydrochloride (15 mg l^−1^); in the degradation process of RhB: 25 mg of catalyst was dispersed in 50 ml of RhB (5 mg l^−1^). The reactor was stirred in the dark for 20 min before irradiation to ensure absorption–desorption equilibrium. During the process, the suspension was kept under stirring to retain the homogeneity of the suspension.

### 2.5 Photoelectrochemical measurements

Photoelectrochemical analysis was carried out in a conventional three electrode cell. In the working cell, the fabricated electrode (a platinum wire) and reference electrodes Ag/AgCl (saturated KCl) were used. The photocatalyst powder was deposited on FTO (F doped tin oxide) glass by electrophoretic deposition. Electrochemical impedance spectroscopy (EIS) measurements were performed at frequencies from 0.1 Hz to 100 KHz and the applied voltage was the open circuit voltage. The electrolyte was Na_2_SO_4_ aqueous solution (0.2 M, pH = 7).

## 3. Results and discussion

### 3.1 Structural and morphological analysis

**Fig. 1:**
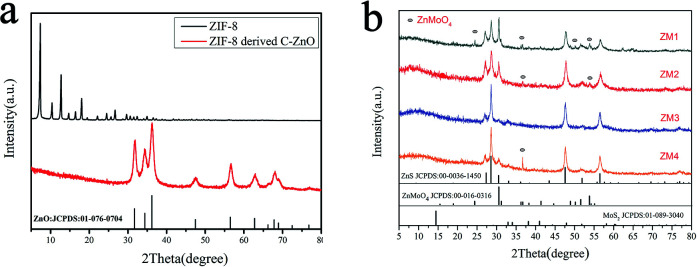
XRD patterns of (a) ZIF-8 and ZIF-8 derived ZnO and (b) ZM1, ZM2, ZM3 and ZM4.

The crystal phases of the samples (ZIF-8 C–ZnO ZM) were first identified by X-ray diffraction and the results are shown in Fig. 1.

As shown in Fig. 1a, the transition from ZIF-8 to C–ZnO can be seen. The XRD pattern of ZIF-8 is consistent with the patterns reported in the literature.^37^ After thermal annealing, the diffraction peaks of ZIF-8 derived C–ZnO located at about 31.738°, 34.380°, 36.217°, 47.487°, 56.535°, 62.777°, 67.865° can be indexed to the (100), (002), (101), (102), (110), (103) and (112) diffraction planes of ZnO (JCPDS no. 01-076-0704), which reflects the successful transformation from ZIF-8 to ZnO.

The XRD patterns of the ZMs are shown in Fig. 1b. Six peaks located at about 26.915°, 28.502°, 30.530°, 47.563°, 51.778°, 56.394° correspond to diffraction from the (100), (002), (101), (110), (103) and (112) crystallographic planes of ZnS (JCPDS no. 00-036-1450) respectively in all samples. The peaks corresponding to ZnMoO_4_ can be observed at 24.302°, 36.620°, 48.844°, 53.715° corresponding to the (110), (120), (022) and (−221) diffraction planes of ZnMoO_4_ (JCPDS no. 00-016-0310) in ZM1, 36.620° and 53.715° corresponding to the (120) and (−221) diffraction planes of ZnMoO_4_ in ZM2, and 36.620° corresponding to the (120) diffraction planes of ZnMoO_4_ in ZM4, and no peaks corresponding to ZnMoO_4_ were observed in ZM3. The peaks located at 33.4° corresponding to MoS_2_ could be observed in ZM3. Unfortunately, no diffraction peaks corresponding to MoS_2_ could be discerned in the other XRD patterns, which may be because of the low content of this material in the three composites. Other than this, we also found that the main diffraction peaks corresponding to MoS_2_ at 37.6° and 57.8° were almost coincident with the diffraction peaks corresponding to ZnMoO_4_ at 36.620° and ZnS at 56.394°, which may also cause the inconspicuous peaks of MoS_2_ in the XRD patterns of these composites, except for ZM3 which did not contain the component of ZnMoO_4_. The presence of MoS_2_ and the composition of the catalysts will be further corroborated by SEM, TEM and XPS images.

**Scheme 1:**
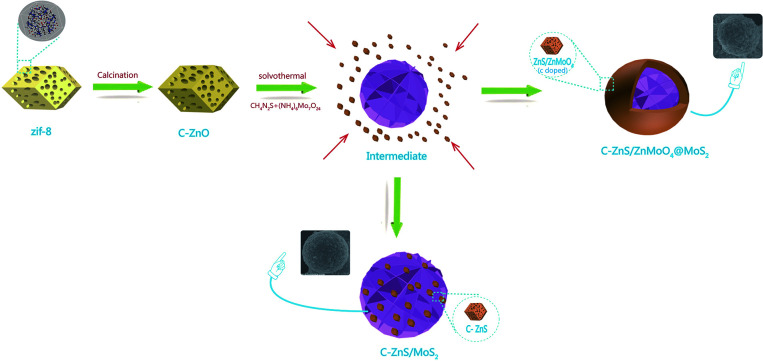
Synthetic procedure of ZIF-8 derived C–ZnS/ZnMoO_4_@MoS_2_ and C–ZnS/MoS_2_.

The synthetic procedure for ZIF-8 derived C–ZnS/ZnMoO_4_@MoS_2_ and C–ZnS/MoS_2_ is shown in Scheme 1. As shown in Scheme 1, ZIF-8 derived C–ZnO was mixed with the raw materials for the synthesis of MoS_2_. During the hydrothermal reaction, when the MoS_2_ is forming from the reaction of thiourea and ammonium molybdate, ZIF-8 derived C–ZnO will also react with thiourea and ammonium molybdate to generate C–ZnS/ZnMoO_4_ or C–ZnS. The differences in the products is because the amount of thiourea will affect the acid–base properties of the solution and this may be the requirement for determining whether or not ZnMoO_4_ can be formed. Since the raw material in the preparation of C–ZnS/MoS_2_ (ZM3) was used in a larger amount than in the preparation of ZnS/ZnMoO_4_@MoS_2_ (ZM1, 2 and 4), the size of MoS_2_ in ZM3 was much larger than that in ZM2, eventually resulting in the different morphologies of ZM2 and ZM3, as shown in Scheme 1.

The morphologies of the composites were investigated by SEM and TEM. Pure flower-like MoS_2_ is shown in Fig. S1.† It should be noted that pure MoS_2_ exhibited a smaller diameter than MoS_2_ in C–ZnS/MoS_2_ or C–ZnS/ZnMoO_4_@MoS_2_, the reason being that, in the process of preparing the catalysts, the addition of C–ZnO will increase the pH of the solution. According to research,^37,38^ in the process of preparing flower-like MoS_2_, an increase in the pH of the solution will accelerate the growth of the diameter of the obtained MoS_2_. The prepared ZIF-8 shown in Fig. S2† has a uniform polyhedral morphology with a diameter of 200–300 nm.

**Fig. 2:**
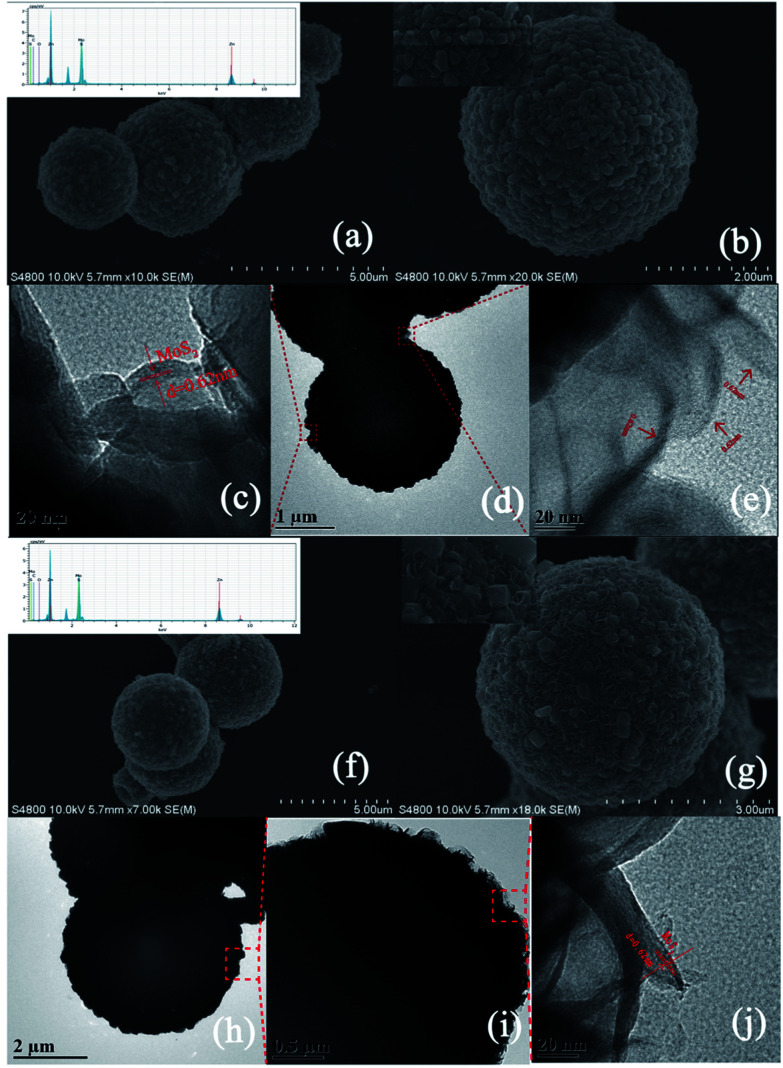
(a) and (b) SEM images and EDS pattern (inset (a)) of ZM2; (d) TEM images of ZM2; (c) and (e) HRTEM images of ZM2; (f) and (g) SEM images and EDS pattern (inset (f)) of ZM3; (h) and (i) TEM images of ZM3; (j) HRTEM images of ZM3.

**Fig. 3:**
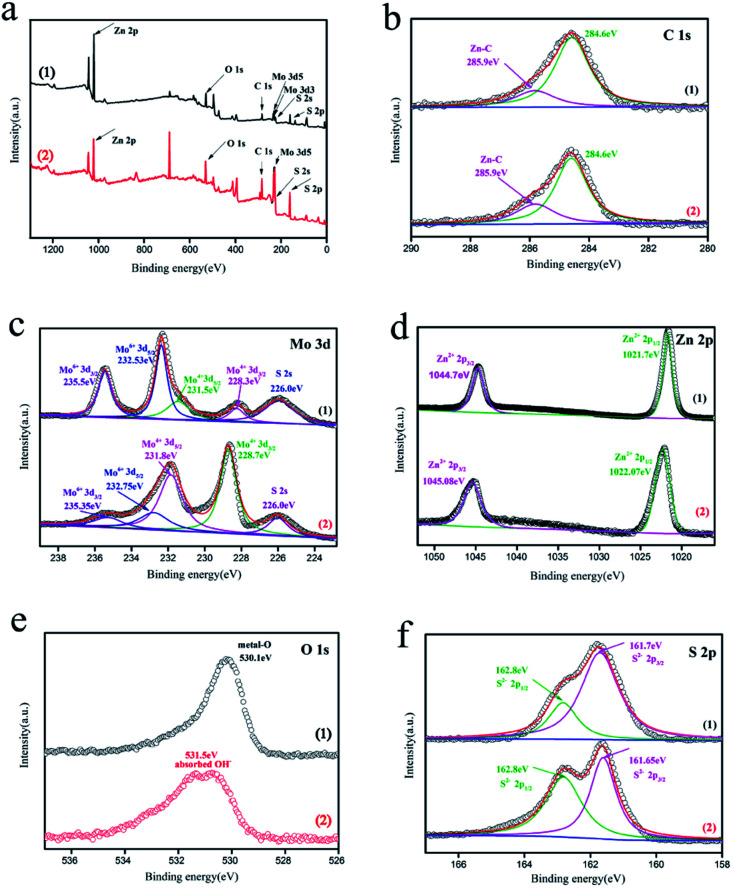
The high resolution XPS spectra of (a) survey spectrum, (b) C 1s, (c) Mo 3d (d) Zn 2p, (e) O 1s, (f) S 2p of (1) C–ZnS/ZnMoO_4_@MoS_2_(ZM2) and (2) C–ZnS/MoS_2_ (ZM3).

After hydrothermal treatment, the sphere-like ZM2 with a rough surface covered with 200–300 nm particles shown in Fig. 2a and b was proved by XRD (Fig. 1b) and XPS (Fig. 3) analysis to be ZIF-8 derived C–ZnS/ZnMoO_4_. We also found that C–ZnS/ZnMoO_4_ approximately retained the polyhedral morphology of ZIF-8 (inset Fig. 1b). The morphologies of MoS_2_ cannot clearly be observed in the SEM images because the surface of ZM2 is covered with nanoparticles of C–ZnS/ZnMoO_4_. However, because MoS_2_ is a flexible kind of material, its nanosheets can pass through the outer layer, which cannot be clearly observed in the SEM images. As shown in Fig. 2c–e, a large number of nanosheets can be found at the surface of ZM2 in the TEM images, and the high-resolution TEM (HRTEM) images (Fig. 2c and e) show clear fringes with a lattice spacing of 0.62 nm, which correspond to the (002) plane of MoS_2_. In addition, EDS analysis shows that selected areas of the ZM2 composites comprise Zn, S, Mo, C and O elements (inset Fig. 2a).

It is worth mentioning that the carbon content can be observed both in the EDS pattern of the ZM2 and ZM3 samples (inset Fig. 2a and f), but no diffraction peaks of carbon were found in the XRD patterns of any of the samples, which indicates that the carbon was successfully doped into the lattice of ZnS/ZnMoO_4_ and ZnS.^39^ This conclusion was also further proved by XPS analysis (Fig. 3). Unlike the composition and morphology of ZM2, the nanoparticles of ZM3 were proved by XRD (Fig. 1b) and XPS (Fig. 3b) analysis to be ZIF-8 derived C–ZnS, and the particles retained the polyhedral morphology of ZIF-8 (inset Fig. 2g) loaded onto the flower-like MoS_2_, as shown in Fig. 2f and g. The nanosheets on the surface of ZM3 can be clearly observed in Fig. 2j and i. The HRTEM (Fig. 2j) images also show fringes with a lattice spacing of 0.62 nm corresponding to the (002) plane of MoS_2_. The EDS analysis shows the ZM3 to have Zn, S, Mo, C and O elements, in which the existence of oxygen might be due to the presence of water molecules absorbed on the surface of ZM3, which was further confirmed by XPS (Fig. 3).

### 3.2 XPS analysis

XPS analysis was carried out to further confirm the chemical composition and surface chemical state of ZM2 and ZM3 (Fig. 3). The characteristic peaks of C, Mo, Zn, O and S elements appear over the whole XPS survey spectrum of the two samples (Fig. 3a) which is consistent with the EDS results. The peaks of the high-resolution spectrum of C 1s are located at 284.6 and 285.9 eV (Fig. 3b). The main peak at 284.6 eV can be assigned to adventitious hydrocarbons and the peak at 285.9 eV can be attributed to Zn–C bonds, which further proves that carbon was doped into the lattice of ZnS/ZnMoO_4_ and ZnS into the C–ZnS/ZnMoO_4_@MoS_2_ and C–ZnS/MoS_2_ samples, respectively.

In Fig. 3c(1), the high resolution spectrum for Mo 3d of ZM2 could be divided into five peaks: the binding energy at approximately 226.0 eV can be attributed to the S 2 s of MoS_2_ and the binding energies of 232.53 and 235.5 eV correspond to the Mo 3d_5/2_ and Mo 3d_3/2_ of Mo^6+^, respectively. The Mo 3d_5/2_ peak reveals that Mo exists in the form of a hexavalent oxide. Another two peaks at 228.3 and 231.5 eV are characteristic of the Mo 3d_5/2_ and Mo 3d_3/2_ of Mo^4+^ in MoS_2_, which further proves the existence of MoS_2_ in the C–ZnS/ZnMoO_4_@MoS_2_ samples (Fig. 2c and e). In Fig. 3c(2), the high resolution spectrum of Mo 3d of ZM3 can also be divided into five peaks. Compared to ZM2 in Fig. 3c(1), the main peaks of ZM3 are located at 228.7 and 231.8 eV, characteristic of Mo 3d_3/2_ and Mo 3d_5/2_ of Mo^4+^, and another two weaker peaks at about 232.75 and 235.35 eV were fitted as Mo^6+^, which might be due to the incomplete decomposition of MoO_4_^2−^, consistent with the XRD results shown in Fig. 1b. The differences between the peak positions might due to the difference in the compositions of the two samples, resulting in a slight shift in the peak position.

As displayed in Fig. 3d(1), two distinct peaks at 1021.7 and 1044.7 eV correspond to Zn 2p_1/2_ and Zn 2p_3/2_, and the slight deviation is also due to the difference in the compositions of the two samples. The characteristic binding energy of 530.1 eV of ZM2 in Fig. 3e(1) for O 1s reveals that O is present in the metal oxide ZnMoO_4_ in C–ZnS/ZnMoO_4_@MoS_2_, and the binding energy at 531.5 eV can be attributed to the chemisorbed or dissociated oxygen or OH species on C–ZnS/MoS_2_. The two peaks at 162.8 and 161.7 eV correspond to the S 2p_1/2_ and S 2p_3/2_ of S^2−^ and the slight difference in the 2p_3/2_ peak of ZM3, which can be found in Fig. 3f(2), is also due to the different compositions of the two samples.

### 3.3 Photocatalytic activity

#### 3.3.1 Photodegradation of TC-H

**Fig. 4:**
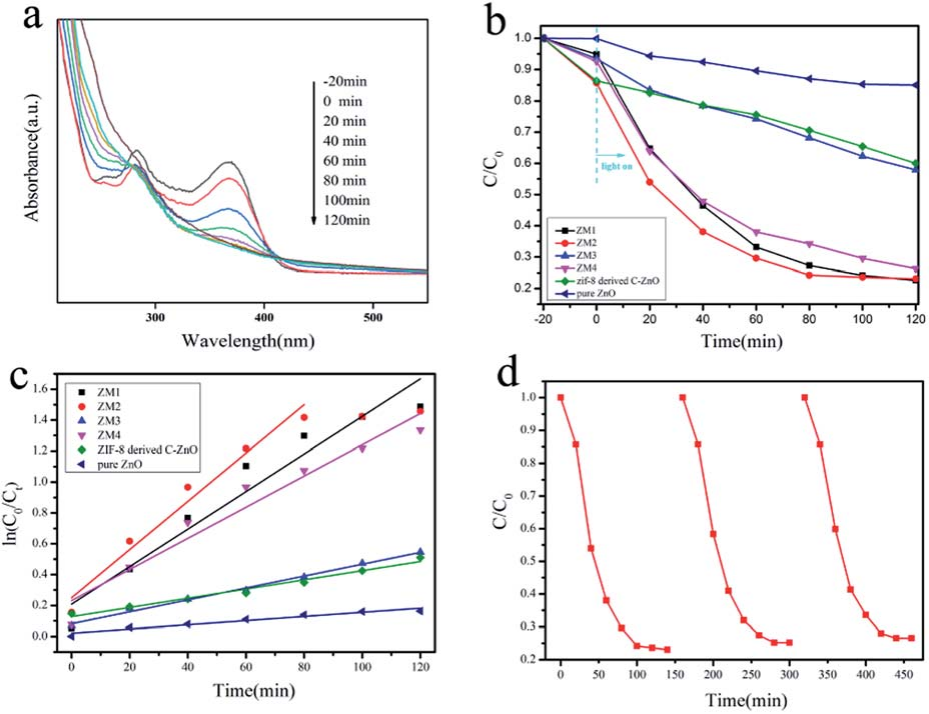
(a) Absorption spectra of TC-H solution collected during the photodegradation of ZM2; (b) the photodegradation efficiency of TC-H (*C*/*C*_0_) as a function of irradiation time over ZM1, ZM2, ZM3, ZM4, ZIF-8 derived C–ZnO and pure ZnO; (c) kinetic linear simulation curves of TC-H degradation with different photocatalysts under full spectrum light irradiation; (d) photocatalytic activities of ZM2 for TC-H degradation with three times of cycling use.

The absorption spectra of TC-H degraded by ZM2 are shown in Fig. 4a. It was found that the characteristic peak at 357 nm decreased rapidly under light irradiation and became barely visible after 60 min of irradiation. When the irradiation time was further increased to 80 min, the absorbance spectra barely changed. The absorption spectra of TC-H solutions degraded by other samples are shown in Fig. S3.† The photocatalytic degradation results of all the samples (ZM1-4) are shown in Fig. 4b. As shown in Fig. 4b, ZIF-8 derived C–ZnO exhibits enhanced catalytic efficiency compared to pure ZnO, which proves the superiority of C–ZnO prepared from ZIF-8 as a precursor in terms of photocatalytic performance compared with pure ZnO. All the ZM samples (ZM1, ZM2, ZM3 and ZM4) showed higher photocatalytic efficiency than ZIF-8 derived C–ZnO because of the heterojunction combination of C–ZnS, C–ZnMoO_4_ and MoS_2_, and such a structure can improve the photodegradation activity of these photocatalysts. Among them, ZM2 exhibited the highest photocatalytic activity for the degradation of TC-H and the ratio *C*/*C*_0_ decreased from 58% to 20% compared to ZIF-8 derived C–ZnO. It is worth noting that the catalytic efficiency of ZM3 was much lower than those of ZM1, ZM2 or ZM4, and only 60% of TC-H could be degraded in 120 minutes under light irradiation. Compared to ZM3, which is only composed of C–ZnS and MoS_2_, ZM1, ZM2 and ZM4 are three-phase catalysts composed of C–ZnS, C–ZnMoO_4_ and MoS_2_. The transfer of photogenerated charge carriers in the three phases is equivalent to increasing the transfer distance of carriers and reducing the recombination of the photogenerated electron–hole pairs. In addition, the reason for the differences in the photodegradation activity of ZM1, ZM2 and ZM4 is the different ratios of C–ZnS, C–ZnMoO_4_ and MoS_2_, due to the different amounts of raw materials added during their preparation.

The reaction kinetics of the TC-H degradation were further studied according to the pseudo-first-order kinetic model shown eqn (1), and the results are shown in Fig. 4c

1 ln(*C*_0_/*C*_*t*_) = *kt*

in which *t* is the reaction time, *k* is the rate constant, and *C*_0_ and *C*_*t*_ are the concentrations of the TC-H solution at times 0 and *t* respectively.

It should be noted that TC-H was completely degraded by ZM2 in 80 minutes (Fig. 4a), so only the first five points were taken to obtain the slope (eqn (1)). As shown in Fig. 4c, the rates of TC-H photodegradation were 0.001, 0.003, 0.012, 0.016, 0.004, and 0.010 min^−1^ for pure ZnO, ZIF-8 derived C–ZnO, ZM1, ZM2, ZM3 and ZM4, respectively. Thus, the ZM2 samples showed excellent performance in the degradation of TC-H. In Fig. 4d, the recycling tests reveal that C–ZnS/ZnMoO_4_@MoS_2_ (ZM2) can be used repeatedly at least three times and the photocatalytic activity remains sufficiently stable without any significant deactivation.

#### 3.3.2 Photodegradation of RhB

**Fig. 5:**
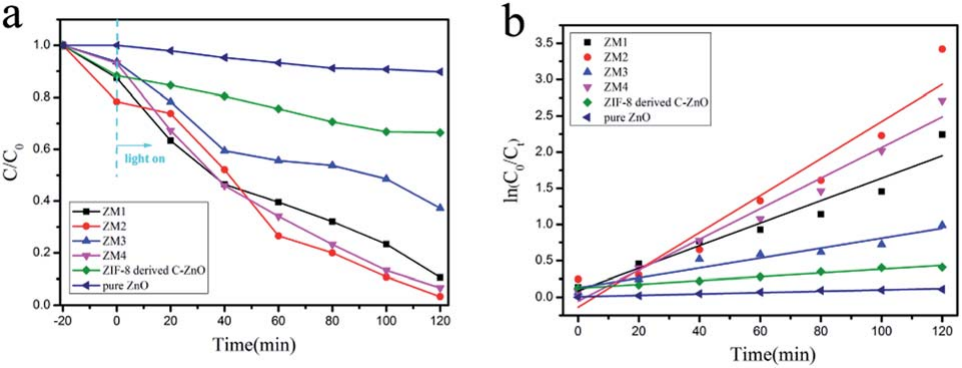
(a) Photodegradation efficiency of RhB (*C*/*C*_0_) as a function of irradiation time over ZM1, ZM2, ZM3, ZM4, ZIF-8 derived C–ZnO and pure ZnO; (b) kinetic linear simulation curves of RhB degradation with different photocatalysts under full spectrum light irradiation.

To further demonstrate their applications in the removal of organic dye from wastewater, the photocatalytic activities of the obtained products were evaluated by measuring the degradation of RhB under full spectrum light irradiation. Fig. 5a indicates that ZM2 exhibits the best photocatalytic performance with an efficiency of 100% compared with the other photocatalysis for the degradation of RhB after irradiation for 120 min. As for the degradation of TC-H, the catalytic efficiencies of ZM1, ZM2 and ZM4 were also better than that of ZM3, and all the ZM (ZM1, ZM2, ZM3 and ZM4) samples exhibited enhanced catalytic efficiency compared to pure ZnO or ZIF-8 derived C–ZnO. As shown in Fig. 5b, the rate constants of different samples are 0.015, 0.026, 0.007 and 0.021 min^−1^ for ZM1-4, respectively.

In order to reveal the underlying reaction mechanism of the enhanced photocatalytic activity of ZM2, the radical and hole generation during the process of photocatalytic degradation of RhB over ZM2 was investigated by adding various scavengers. As shown in Fig. S4,† ammonium oxalate (AO) was added as an h^+^ scavenger, *p*-benzoquinone (PBQ) as an ·O_2_^−^ scavenger and *tert*-butanol (*t*-BuOH) as an ·OH scavenger. It was found that there was a dramatic decrease in the photocatalytic activity when PBQ was added, indicating that ·O_2_^−^ plays an important role in the photocatalytic degradation process. In addition, the photodegradation rate of RhB was also suppressed when AO and *t*-BuOH were added, suggesting that h^+^ and ·OH participate in the photodegradation process of RhB.

### 3.4 Optical analysis

**Fig. 6:**
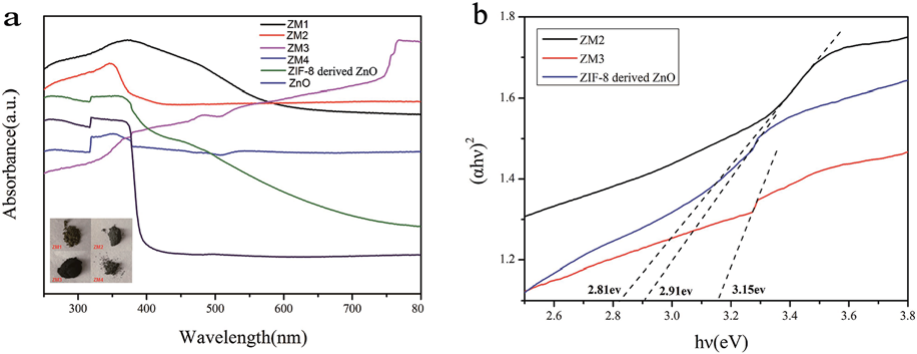
(a) UV-vis diffuse reflection spectra (inset: photographs of ZM1-4) and (b) (*αhv*)^2^*vs. hv* curves of different samples.

Fig. 6 reveals the UV-vis diffuse reflection spectra of pure ZnO, ZIF-8 derived C–ZnO and ZM1-4. As shown in Fig. 6a, the main absorption intensity of pure ZnO occurs at *ca.* 390 nm and the edge of absorption is very low. In contrast to that, the absorption values of ZIF-8 derived C–ZnO extend into the visible light region, which confirms that it can harvest visible light effectively.

It is obvious that using ZIF-8 as a precursor to prepare catalysts has more advantages compared to using pure ZnO as a photocatalyst. After the introduction of MoS_2_ and transformation of ZIF-8 derived C–ZnO to C–ZnS/ZnMoO_4_ and C–ZnS, the absorption peaks of the ZM composite materials were red shifted and the light absorption was obviously enhanced compared to that of ZIF-8 derived C–ZnO. The enhanced absorption intensities of these materials are also consistent with the colors of the catalyst powders (inset Fig. 6a) which will be beneficial for their photodegradation activities.

On the basis of the Kubelka–Munk equation, the band gap energy of a semiconductor can be calculated using the following formula eqn (2)

2 (*αhv*)^2^ = (*hμ* − *E*_g_)

where *h*, *μ*, *E*_g,_ and *α* denote Plank's constant, the frequency of the light, the band gap energy, and the absorbance, respectively.^40^ As shown in Fig. 6b, the *E*_g_ of ZIF-8 derived C–ZnO is estimated to be 2.91 eV, the *E*_g_ of ZM2 to be 2.81 eV, and the *E*_g_ of ZM3 to be 3.15 eV, which were all calculated based on the above formula.

### 3.5 Photoelectrochemical analysis

**Fig. 7:**
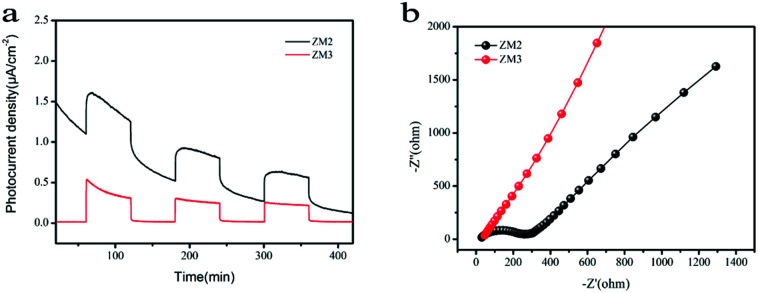
(a) Photocurrent spectra of as-synthesized samples under UV-vis light irradiation; (b) EIS Nyquist plots of the samples under dark condition.

As shown in Fig. 7a, a fast and steady photocurrent response was detected for each light-on and light-off period over ZM2 and ZM3. The ZM2 samples show a significant enhancement in photocurrent density compared with that of the ZM3 samples, which further confirms the efficient transfer of photo-induced electrons of ZM2.

Electrochemical Impedance Spectroscopy (EIS) measurements were also carried out to figure out the interface charge transport behavior of ZM2 and ZM3. As shown in Fig. 7b, the radius of the arc on the EIS Nyquist plot reflects the reactor rate occurring at the electrode surface. The lower arc radius of ZM2 with respect to ZM3 demonstrates a faster interfacial transfer and more effective separation of photo-induced pairs occurring in the composites. These results are well consistent with the above-mentioned photocurrent tests. Thus, the photocurrent spectra and EIS Nyquist plots confirm the superior separation efficiency of the photo-induced electrons and holes and efficient charge transfer in ZM2, which are responsible for the enhanced photocatalytic activity.

The separation efficiency of the charge carriers is also shown in the photoluminescence (PL) analysis. As shown in Fig. S5,† the main emission peak is observed at around 623 nm. The ZM3 sample exhibits a lower PL intensity than ZIF-8 derived C–ZnO, and ZM2 shows the lowest emission peak among the C–ZnO, ZM3 and ZM2 samples, which proves that the construction of the heterojunction structure effectively inhibits photo-induced electron–hole pair recombination and the separation rate of the electron–hole pairs of ZM2 is better than that of ZM3, which is consistent with the results of the photoelectrochemical analysis.

### 3.6 Possible mechanism

**Scheme 2:**
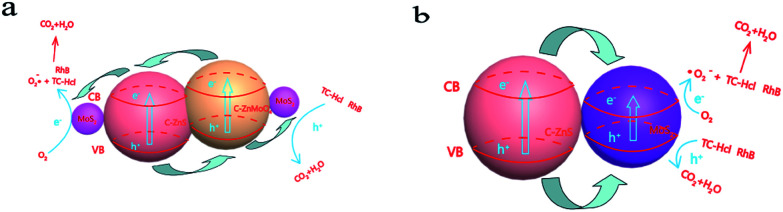
(a) Schematic illustration of the charge transfer in the C–ZnS/ZnMoO_4_@MoS_2_ composites and (b) C–ZnS/MoS_2_ composites under full spectrum light irradiation.

On the basis of the experimental results and previous studies, a possible mechanism for the enhanced photoactivity of TC-H and RhB degradation can be proposed. As shown in Scheme 2a, under the excitation of light, C–ZnS, C–ZnMoO_4_ and MoS_2_ will all be excited at the same time, and the electrons (e^−^) in the valence band (VB) are easily photoexcited to the conduction band (CB), leaving holes (h^+^) in the VB.^41^ The difference between the CB and VB edge potentials of these semiconductors^42,43^ allows the electrons to first transfer from the CB of C–ZnMoO_4_ to the CB of C–ZnS; after that, all the electrons on the CB of C–ZnS will continue to transfer to the CB of MoS_2_. During the process, the holes leaving the VB of C–ZnS first move in the opposite direction to the electrons to the VB of C–ZnMoO_4_, which could result in effective electron–hole separation and the holes will finally transfer to the VB of MoS_2_. It is harder for photogenerated electrons and holes to recombine in the indirect band gap of the MoS_2_ nanosheets. Thus, the lives of photogenerated electrons and holes are prolonged, which results in enhanced photocatalytic activity. And, it should be noted that the C-doping of the structure can improve the visible-light response, which can also enhance the photocatalytic activity.

C–ZnS/MoS_2_ is a two-phase catalyst, so the transfer of electrons and holes only occurs in two phases, which may decrease the separation of the electron–hole pairs and increase the recombination of the photo excitation pairs. Compared to C–ZnS/ZnMoO_4_@MoS_2_, C–ZnS/ZnMoO_4_@MoS_2_ is a three-phase catalyst consisting of C–ZnS, C–ZnMoO_4_ and MoS_2_, so the contact between C–ZnS and C–ZnMoO_4_ will be closer and intersecting compared to the physical contact of general heterojunction photocatalysts, because they were prepared from ZIF-8 derived C–ZnO at the same time using a direct one-pot route. The longer transfer steps and closer contact of the catalyst can facilitate the separation of electron–hole pairs through closer contact between the different semiconductors and enhance the photocatalytic performance of C–ZnS/ZnMoO_4_@MoS_2_.

## 4. Conclusion

In summary, a series of ZIF-8 derived C–ZnS/ZnMoO_4_@MoS_2_ and C–ZnS/MoS_2_ catalysts were successfully produced by an efficient and facile one-pot strategy. The as-prepared ZIF-8 derived C–ZnS/ZnMoO_4_@MoS_2_ and C–ZnS/MoS_2_ show enhanced photocatalytic activity in the photocatalytic degradation of TC-H and RhB compared to ZIF-8 derived C–ZnO or pure ZnO. C–ZnS/ZnMoO_4_@MoS_2_ also shows better photocatalytic capability over C–ZnS/MoS_2_. The enhanced photocatalytic capability is due to the C-doped structure and the heterojunction structure, which improve the visible-light response and facilitate the separation of photogenerated electron–hole pairs and reduce the recombination of photogenerated charge carriers. We believe that such novel ZIF-8 derived C–ZnS/ZnMoO_4_@MoS_2_ and C–ZnS/MoS_2_ heterojunction structures can potentially be developed into a new-type of photocatalyst.

## Conflicts of interest

The authors declare no conflict of interest.

## Supplementary Material

RA-009-C9RA06591A-s001
