# Supplementary material for: Constructing ZIF-8 derived C–ZnS/ZnMoO4@MoS2 and C–ZnS/MoS2 nanocomposites using a simple one-pot strategy to enhance photocatalytic degradation activity
Source: RSC Adv. 2019 Oct 31;9(60):35189–96. doi: 10.1039/c9ra06591a (PMC9074736; doi:10.1039/c9ra06591a)
Supplement: RA-009-C9RA06591A-s001 [file RA-009-C9RA06591A-s001.pdf]

Electronic Supplementary Material (ESI) for RSC Advances.
This journal is © The Royal Society of Chemistry 2019

Supporting information

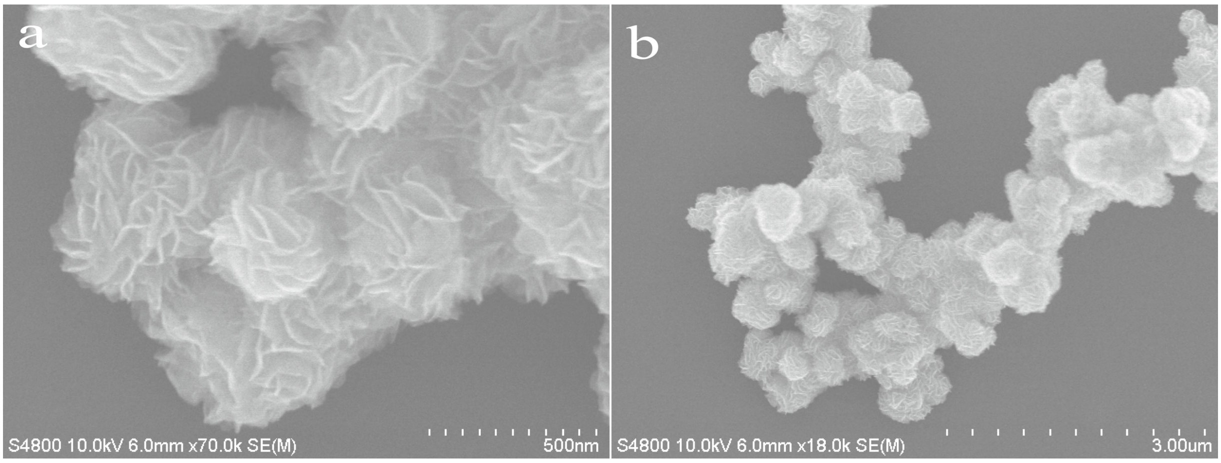

FigS1. SEM images of pure MoS_2 produced by thiourea and ammonium molybdate

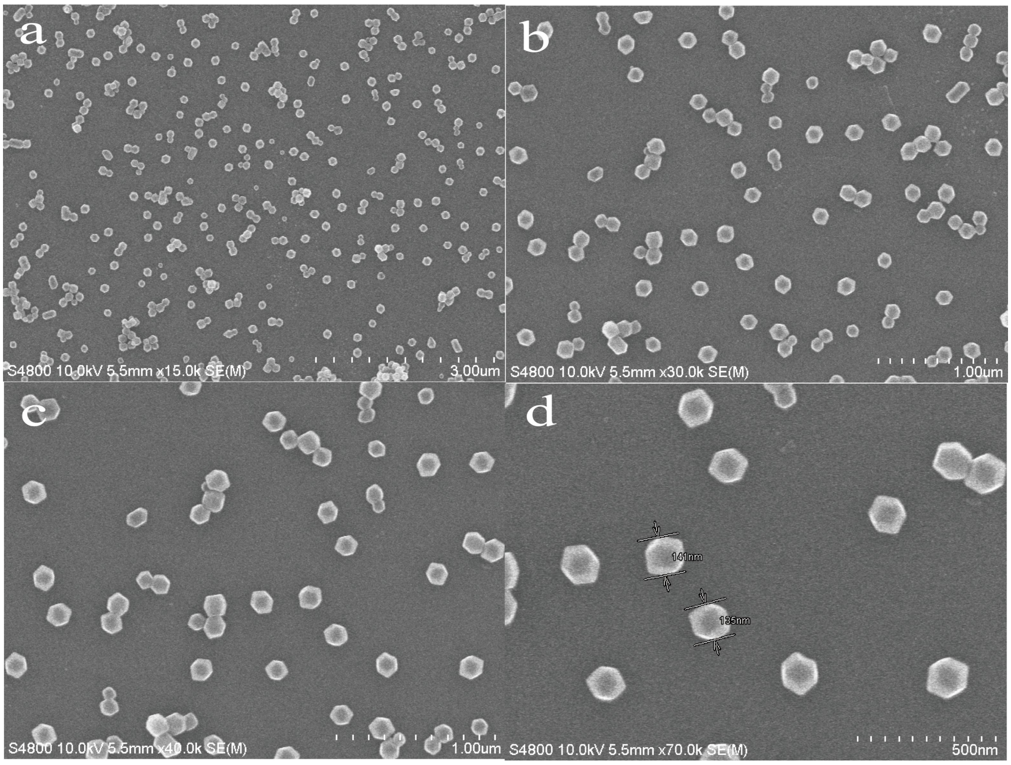

FigS2. SEM images of ZIF-8

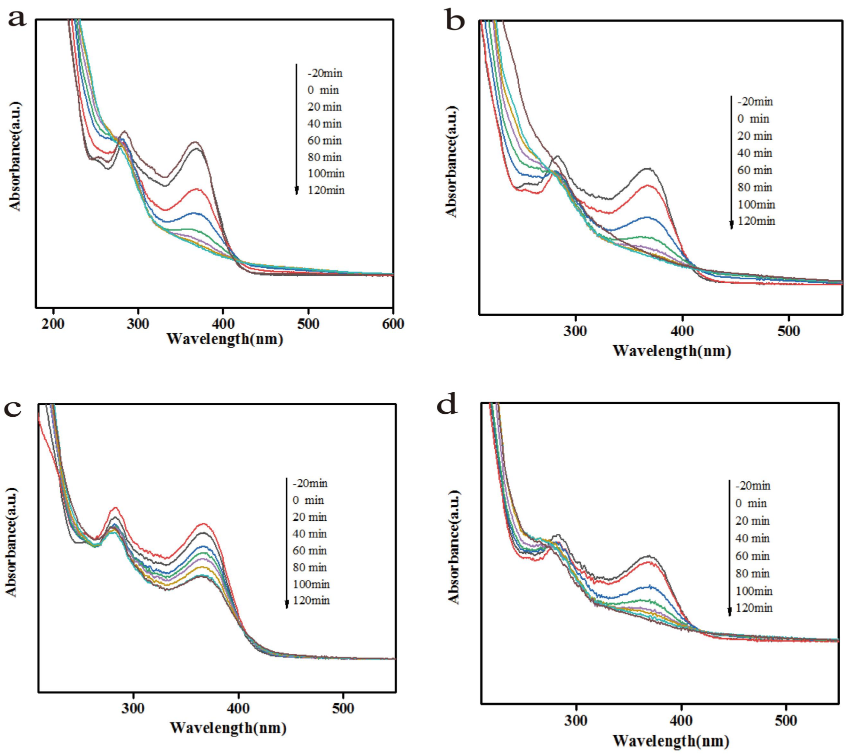

FigS3. Absorption spectra of TC-H solution collected during photodegradation of a)ZM1 b)ZM2 c)ZM3 d)ZM4
